# Analysis of selected polymorphisms in FOXP3 gene in a cohort of Egyptian patients with schizophrenia

**DOI:** 10.1186/s43141-022-00371-y

**Published:** 2022-05-31

**Authors:** Maged Mostafa, Aya Ahmed Fathy, Mohamed Elwasify, Maha Abdelsalam

**Affiliations:** 1 Clinical Pathology Department, Faculty of Medicine, Mansoura University, Mansoura, Egypt; 2 Public Health and Community Department, Faculty of Medicine, Mansoura University, Mansoura, Egypt; 3 Psychiatry Department, Faculty of Medicine, Mansoura University, Mansoura, Egypt; 4 Immunology Department, Egypt Center for Research and Regenerative Medicine (ECRRM), Cairo, Egypt

**Keywords:** Schizophrenia, FOXP3, Egyptian, SNPs, Treg

## Abstract

**Background:**

Schizophrenia is a chronic mental disorder with different symptoms. The environmental and genetic factors are suggested to be the etiology of schizophrenia. However, the exact cause and pathogenesis of schizophrenia are still unclear. Different studies suggested that the immune system may have a role in schizophrenia. A genetic study found a relation between the disease and the HLA region on the sixth chromosome. Regulatory T cells (Treg) have a role in the regulation of immune response, especially the balance between TH1 and TH2 cells. The FOXP3 protein is a key regulator for Treg cell’s functions. FOXP3 is a transcriptional factor, and its gene is present on the short arm of the X chromosome. The selected SNPs present in the promoter region which act as binding sites for transcriptional factors. This study investigated FOXP3 gene polymorphisms (rs3761548, rs3761549, and rs2232365) in Egyptian patients with schizophrenia. There are no previous studies about the association of FOXP3 gene polymorphisms with schizophrenia. The three selected single-nucleotide polymorphisms (SNPs) were investigated using polymerase chain reaction-restriction fragment length polymorphism (PCR-RFLP) for 125 schizophrenia patients and 160 healthy controls. The Positive and Negative Syndrome Scale (PANSS) was used to evaluate patients with schizophrenia.

**Results:**

No significant associations were found between schizophrenia patients and healthy controls for the alleles and genotypes of the selected SNPs (*P*-value > 0.05). However, a significant association with ACC and ATC haplotypes was detected (*P*-value 0.001). No significant association was detected between the PANSS score and any of the studied SNPs.

**Conclusion:**

The ATC haplotype of rs2232365, rs3761549, and rs3761548 could be considered a risk factor for schizophrenia in Egyptian patients.

**Supplementary Information:**

The online version contains supplementary material available at 10.1186/s43141-022-00371-y.

## Background

Schizophrenia is a chronic psychiatric disorder characterized by different types of symptoms, positive symptoms (hallucinations, etc.), negative symptoms (lack of motivation, etc.), and cognitive functions defects [2, 24]. The prevalence of schizophrenia is nearly 1% worldwide. In Egypt, a national survey of mental disorders performed over 14,640 adults between the age of 18 and 64 showed that the prevalence of psychotic disorders including schizophrenia was 0.19% [19]. Schizophrenia markedly affects and disturbs the mental function of the patient and causes suffering for the patients and their families [47]. The manifestations of schizophrenia have been determined for decades, but the exact etiology and pathophysiology are still unclear [39, 40].

There are growing pieces of evidence that support the role of the immune system in the development of schizophrenia [5, 21, 31]. Many genetic studies supported the relationship between the immune system and schizophrenia [6, 22, 30]. Recent genetic studies revealed the relationship between the HLA (human leucocyte antigen) region on chromosome no. 6 and the development of the disease [43]. Another interesting finding is the relationship between autoimmune disorders and schizophrenia. The incidence of autoimmune diseases is more in schizophrenia patients than in the general population. Some researchers found that the incidence of schizophrenia increases to 45% in patients with a history of autoimmune diseases [12]. Another study from Taiwan supported this finding [8].

Regulatory T cells (Treg) are valuable players in maintaining peripheral tolerance and prevent immune system activation against self-antigens [9]. Disruption of Treg functions leads to disturbance in the balance between T helper 1 cells (TH1) and T helper 2 cells (TH2) and the development of different immune and autoimmune disorders. Although this pivotal role in immune system regulation, limited numbers of researchers investigated the role of Treg in schizophrenia pathogenesis [42]. A recent study showed that Treg cells were increased in schizophrenic patients under treatment [28]. Another study showed that Treg cells were increased in patients with recent-onset schizophrenia [11].

The forkhead box protein 3 (FOXP3) is a transcription factor expressed by CD4+/CD25+/FoxP3+ Treg and is essential for Treg development and function. FOXP3 gene is located on the short arm of the X chromosome, contains 12 exons and 11 introns [50]. Genetic mutations in the Foxp3 gene lead to disturbance in the regulatory functions of Treg cells and the development of different immunological disorders like systemic lupus erythematosus [29], multiple sclerosis [10], and allograft rejection [38].

Several studies have assessed the role of immune system regulatory genes in schizophrenia pathogenesis. Consequently, in the current study, we focused on three FOXP3 gene single-nucleotide polymorphisms (SNPs): rs3761548 C/A, rs3761549 C/T, and rs2232365 A/G and their potential association with schizophrenia. All selected SNPs are located in the promoter region of the FOXP3 gene, so they may affect the transcriptional activity of the gene [41]. To the best of our knowledge, there are no previous studies regarding the association of FOXP3 gene polymorphisms and schizophrenia disorder.

## Subjects and methods

### Patients and controls

This work is a case-control study on a cohort of unrelated Egyptian patients with schizophrenia. There were 125 patients with schizophrenia (71 females and 54 males) with age mean (32.45 ± 9.167) years. All patients were examined by two physicians and diagnosed according to the *Diagnostic Statistical Manual of Mental Disorders-IV* (DSM-IV) criteria [13]. Patients were recruited from the Department of Psychiatry. Exclusion criteria include other psychiatric disorders, mental retardation, brain trauma, brain tumors, drug or alcohol addiction, significant medical and/or neurological disorders, and current infections. A Positive and Negative Syndrome Scale (PANSS) was used to assess the severity of symptoms [27]. Other data were collected from medical records or with patient interviews, including age, age of onset, smoking habit, marital status, medication, and laboratory investigations.

The control group comprising 160 healthy unrelated individuals with age mean (26.79 ± 5.89) years was recruited from volunteer donors in the blood bank. Written consents were obtained from both patients and healthy controls after getting approved by the local health committee Faculty of Medicine with approval number (R.20.07.942).

### Genotyping

A 3 ml of venous blood was collected from both patients and control on the EDTA tube using an aseptic venipuncture technique. The samples were reserved at −20 °C until use. DNA extraction was done using the GeneJET Whole Blood Genomic DNA Purification Mini Kit (cat number: K0781; Thermo Scientific, Lithuania, EU). The integrity of purified DNA was tested using 1% agarose gel electrophoresis stained with ethidium bromide. In the genotype of selected SNPs using Polymerase Chain Reaction-Restriction Fragments Length Polymorphism (PCR-RFLP), the primers, restriction enzymes, and fragments size were shown (Table 1).

**Table 1:** Primers sequence, restriction enzymes, and fragments size of selected SNPs

Position	Primer sequence	PCR product	Enzyme restriction	Product
−3279C/A (rs3761548)	Forward: 5′-CTTAACCAGACAGCGTAGAAGG-3′	399 bp	PstI	C: 188 bp + 211 bp
	Reverse: 5′-CATCATCACCACGCTCTG-3′			A: 399 bp
−2383C/T (rs3761549)	Forward: 5′-GCCTGGCACTCTCAGAGCTT-3′	942 bp	BsrI	C: 528bp and 377bp
	Reverse: 5′-GTCTGTGGAGGCTCCGAACA-3′			T: 213bp and 164bp
−924A/G (rs2232365)	Forward: 5′-GTGGAGGGCTTTCAAGGT-3′	682 bp	Tth111I	A: 682 bp
	Reverse: 5′-TCCTCGGAGTCCTATTTTGCC-3′			G: 187 bp + 495 bp

The PCR final volume was 25 μl [5 μl DNA extract, 0.5 μh of each primer, 15 μl master mix (Taq Green 2×, Thermo Fisher Scientific, MA, USA), 4 μl H2O remaining volume]. The reaction was done at thermocycler PTC-100 (Bio-Rad, Hercules). For rs3761548 [41], the cycling parameters were initial denaturation for 5 min at 94 °C followed by 30 cycles for 30 s, annealing at 68 °C for 30 s, extension at 72 °C for 30 s, and a final extension at 72 °C for 10 min. For rs3761549 [46], the cycling parameters were initial denaturation at 95 °C for 3 min, followed by 30 cycles for 30 s, annealing at 60 °C for 30 s, the extension for 1 min at 72 °C, and a final extension at 72 °C for 10 min. For rs2232365 [41], the cycling parameters were initial denaturation for 5 min at 95 °C followed by 30 cycles for 30 s, annealing at 53.3 °C for 30 s, the extension for 45 s at 72 °C, and final extension for 5 min at 72 °C. After PCR, the RFLP was done using specific restriction enzymes for each SNP. PstI (Thermo Fisher Scientific, Cat No. ER0611) was used for rs3761548 with reaction volume of 30 μl (10 μl PCR product, 17 μl nuclease-free water, 2 μl 10× fast digest green buffer, and 1 μl of restriction enzyme) and then incubation at 37 °C for 2 h and chemically inactivated by adding EDTA at PH 8.0. BsrI (Thermo Fisher Scientific, Cat No. ER0881) was used for rs3761549, the same reaction conditions as before, but the incubation for 1 h and thermally inactivated at 80 °C for 20 min. Tth111I (Thermo Fisher Scientific, Cat No. ER1331) was used for rs2232365, also the same conditions except incubation for 4 h at 37 °C and inactivation at 80 °C for 20 min. DNA fragments were resolved at 2.5% agarose gel. A total of 10% of samples were randomly selected for a rerun to check genotyping errors; no differences were detected between the original run and the rerun.

### Statistical analysis

All data were presented using the Excel program (Microsoft Office, 2016). Statistical analysis was done by SPSS version 25 (Statistical Package for Social Science) (SPSS, IBM, Chicago, III, USA). A chi-square test was used to make *a comparison between cases and controls*. Quantitative data were presented as mean and standard deviation, while qualitative data were presented as frequency. A one-way ANOVA test was used to find the relation between nonparametric data. The Hardy–Weinberg equilibrium fitness in both female patients and controls was estimated using the chi-square test. The odds ratio and 95% confidence interval (CI) were calculated, and a *p*-value of less than 0.05 was considered statistically significant. Haploview program version 4.2 was used to detect haplotypes and linkage disequilibrium (LD) [4]. Pairwise FST statistics were calculated using GenAlEx version 6.5 (Genetic Analysis in Excel) [36].

## Results

Table 2 shows demographic, clinical, and laboratory data for studied schizophrenia patients. All cases and control were unrelated to each other and randomly selected from Dakahlia governorate in the delta region in Egypt. The Hardy–Weinberg equilibrium (HWE) is for female patients and controls but not to males as they carry only one copy on the X chromosome. The *p*-value was used to express HWE. The *p*-value for all studied SNPs was more than 0.05, i.e., all SNPs were independent; in other words, they are in HW equilibrium. HWE is an important tool in studying the genetics of a population. It is used to estimate the number of variants (homozygous and heterozygous) based on the allele frequency in not evolving populations; any deviations from HWE indicate genotyping error which may be due to marked heterozygote excess [1].

**Table 2:** Demographic, clinical, and laboratory data

Age years (mean ± SD)	32.45 ± 9.167
Gender: male/female	54/71
Duration of illness: years (mean ± SD)	9.48 ± 6.875
Family history: no/yes	78/47
Smoking: no/yes	49/76
Marital status: single/married/divorced	71/34/20
Antipsychotic medication: no/yes	22/103
Mood stabilizer: no/yes	93/32
Antidepressant: no/yes	77/48
Diabetes/hypertension: no/yes	117/8
Positive scale 7–49 (mean ± SD)	16.66 ± 4.675
Negative scale 7–49 (mean ± SD)	19.30 ± 15.939
General psychopathology 16–112 (mean ± SD)	55.54 ± 27.286
Total score	91.48 ± 30.815
HB g/dl (mean ± SD)	15.362 ± 15.546
Wbcs 10^9^/l (mean ± SD)	7.215 ± 1.824
Platelets 10^9^/l (mean ± SD)	228.79 ± 87.216
ALT U/l (mean ± SD)	25.47 ± 14.229
Glucose mg/dl (mean ± SD)	93.98 ± 19.122
Creatinine mg/dl (mean ± SD)	0.980 ± 0.4879
Cholesterol mg/dl (mean ± SD)	190.55 ± 133.152

*SD* standard deviation

Table 3 shows the distribution of alleles and genotypes for studied SNPs in both males and females. No statistically significant differences were observed between healthy controls and schizophrenia patients for any of the studied SNPs in both males and females (*p*-value > 0.05).

**Table 3:** Distribution of the FOXP3 alleles and genotypes in schizophrenia patients and healthy controls

Gender	Foxp3 polymorphism	Case (*N* = 125) *N* (%)	Control (*N* = 160) *N* (%)	OR (95% *CI*)	*P*-value
Female	rs3761548 (C/A)				
	AA	3 (4.2)	5 (5.4)	0.77 (0.18–3.36)	0.73*
	AC	22 (31.0)	33 (35.5)	0.82 (0.42–1.58)	0.55
	CC	46 (64.8)	55 (59.1)	1.27 (0.67–2.41)	0.46
	A	28 (19.7)	43 (23.1)	0.82 (0.48–1.39)	0.46
	C	114 (80.3)	143 (76.9)	1.22 (0.72–2.09)	0.46
	HWE	0.80	0.77		
Male	A	8 (14.8)	6 (9.0)	1.77 (0.57–5.45)	0.32
	C	46 (85.2)	61 (91.0)	0.57 (0.18–1.74)	0.32
Female	rs3761549 (C/T)				
	TT	2 (2.8)	2 (2.2)	1.31 (0.18–9.59)	0.78*
	CT	29 (40.8)	38 (40.9)	0.99 (0.53–1.87)	1.00
	CC	40 (56.3)	53 (57.0)	0.97 (0.52–1.82)	0.93
	T	33 (23.2)	42 (22.6)	1.04 (0.62–1.75)	0.89
	C	109 (76.8)	144 (77.4)	0.96 (0.57–1.62)	0.89
	HWE	0.77	0.77		
Male	T	18 (33.3)	12 (17.9)	2.29 (0.98–5.32)	0.054
	C	36 (66.7)	55 (82.1)	0.44 (0.19–1.01)	0.054
Female	rs2232365 (A/G)				
	AA	7 (9.9)	10 (10.8)	1.02 (0.37–2.81)	0.97
	AG	31 (43.7)	46 (49.5)	0.79 (0.42–1.47)	0.46
	GG	33 (46.5)	37 (39.8)	1.10 (0.58–2.08)	0.76
	A	45 (31.6)	66 (35.5)	0.84 (0.53–1.34)	0.47
	G	97 (68.3)	120 (64.5)	1.18 (0.75–1.89)	0.47
	HWE	0.68	0.65		
Male	A	24 (44.4)	32 (47.8)	0.88 (0.43–1.79)	0.72
	G	30 (55.6)	35 (52.2)	1.14 (0.57–2.35)	0.72

*OR* odds ratio, 95% *CI* 95% confidence interval, *HWE* Hardy–Weinberg equilibrium

*Fisher’s exact

Table 4 shows the frequency of FOXP3 haplotypes in both schizophrenia patients and healthy control. The GCC haplotype recorded the highest frequency in both studied groups. The ATC haplotype was the least in the cases, while the ACC haplotype was the least in controls. The comparison between haplotype frequency in both patients and controls revealed a significant difference for both ACC and ATC haplotypes (*p*-value = 0.001).

**Table 4:** Distribution of the foxp3 haplotypes in schizophrenia patients and healthy controls

rs2232365 (A/G)	rs3761549 (C/T)	rs3761548 (C/A)	Control	Case	*P*-value
G	C	C	0.638	0.612	0.5854
A	T	A	0.168	0.181	0.7107
A	C	C	0.086	0.162	0.0181
A	T	C	0.093	0.032	0.0071

Table 5 shows the relationship between different genotypes and schizophrenia symptomatology represented as the PANSS score. PANSS is composed of 7 positive symptoms, 7 negative symptoms, and 16 general psychopathological symptoms. Each item from these 30 items takes a score from 1 to 7. No significant association with any of the items of PANSS score of schizophrenia and SNPs genotypes (*p*-value > 0.05) was observed.

**Table 5:** Association of the FOXP3 SNPs with the PANSS score of schizophrenia

	N	Positive scale 7–49			Negative scale 7–49			General psychopathology 16–112			Total score		
		Mean	SD	*p*-value	Mean	SD	*p*-value	Mean	SD	*p*-value	Mean	SD	*p*-value
rs3761548													
aa	11	16.18	5.811	0.938	18.36	4.130	0.902	53.82	29.576	0.916	88.36	27.688	0.934
ac	22	16.77	3.963		18.14	5.240		57.59	31.556		92.50	31.324	
cc	92	16.68	4.735		19.68	18.368		55.25	26.214		91.61	31.334	
rs3761549													
aa	20	17.50	6.856	0.607	17.65	5.363	0.757	65.80	27.922	0.186	100.9	28.772	0.302
ac	29	16.14	4.095		18.21	4.996		53.38	29.247		87.72	29.254	
cc	76	16.63	4.204		20.14	20.038		53.66	26.089		90.43	31.785	
rs2232365													
aa	31	16.45	6.334	0.835	17.48	4.836	0.462	62.45	26.120	0.237	96.38	27.840	0.346
ac	31	16.35	3.702		17.52	5.098		51.26	28.428		85.12	27.130	
cc	63	16.90	4.188		21.06	21.859		54.24	27.077		92.20	33.694	

*SD* standard deviation

Table 6 compares the results of allele frequencies of studied SNPs in healthy control and the five populations in the 1000 genomes project (African, American, East Asian, European, and South Asian). Similarities were found between this current study and the other populations except for rs3761548; a diversity was found with the European population (*Fst* = 0.103).

**Table 6:** Comparison of the studied SNPs genetic variability between healthy control subjects in the current study and main ethnic groups in the 1000 genome project

SNP ID	Population	N	MA	MAF	Ho	He	Fst versus current
rs3761548	Current study	160	A	0.173	0.197	0.279	
	All populations	2504	A	0.237	0.159	0.361	0.007
	African	661	A	0.047	0.045	0.092	0.039
	American	347	A	0.292	0.193	0.416	0.021
	East Asia	504	A	0.166	0.139	0.275	0.000
	European	503	A	0.478	0.282	0.798	0.103
	South Asia	489	A	0.280	0.184	0.405	0.017
rs3761549	Current study	160	T	0.185	0.208	0.282	
	All populations	2504	T	0.144	0.111	0.248	0.001
	African	661	T	0.023	0.018	0.050	0.079
	American	347	T	0.084	0.075	0.153	0.030
	East Asia	504	T	0.190	0.163	0.309	0.000
	European	503	T	0.121	0.103	0.213	0.013
	South Asia	489	T	0.330	0.219	0.441	0.019
rs2232365	Current study	160	A	0.345	0.274	0.450	
	All populations	2504	A	0.410	0.215	0.483	0.005
	African	661	A	0.187	0.154	0.295	0.062
	American	347	A	0.546	0.222	0.497	0.017
	East Asia	504	A	0.641	0.236	0.460	0.056
	European	503	A	0.401	0.262	0.483	0.000
	South Asia	489	A	0.383	0.223	0.473	0.001

*N* number within each population, *MA* minor allele, *MAF* minor allele frequency, *Ho* observed heterozygosity, *He* expected heterozygosity, *uHe* unbiased expected heterozygosity, *Fst* fixation index

**Fig. 1:**
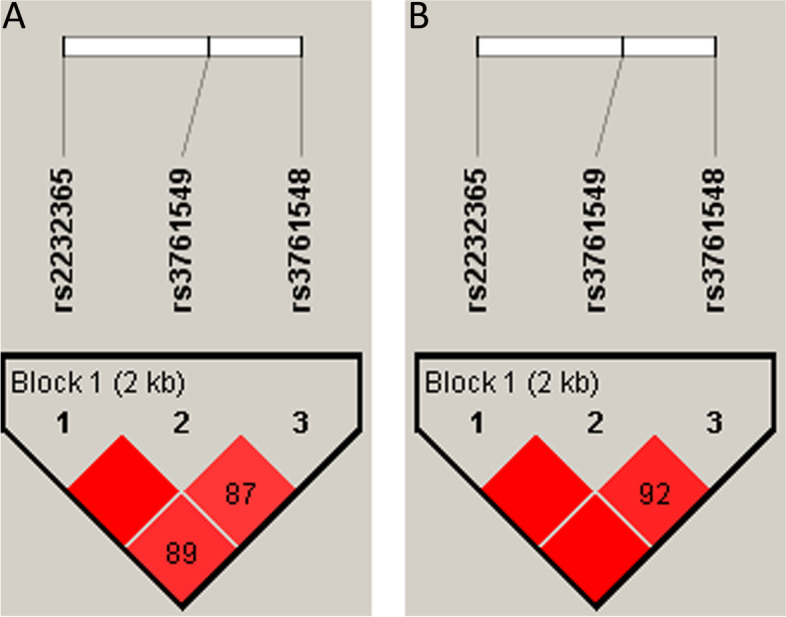
Shows the LD between the studied SNPs of FOXP3 **A** cases and **B** controls. The colored squares represent the numerical estimation of the D’ value. The square without numbers of D’ value is 1

Linkage disequilibrium was examined for studied FOXP3 SNPs. D’ value for LD between rs2232365 and rs3761549 was 1.0 in cases and controls, and the same result was obtained for LD between rs2232365 and rs3761548 in controls; this means complete LD, i.e., the 2 SNPs are not separated by recombination, while in cases, it was 0.899. For rs3761549 and rs3761548, the D’ value for LD was 0.877 and 0.923 for cases and controls, respectively (Fig. 1).

## Discussion

In the present study, we investigated the association of FOXP3 gene polymorphisms and schizophrenia for the first time. All individuals in this study were Egyptian from the Dakahlia governorate, delta region, Egypt. No statistically significant differences were observed between schizophrenia patients and healthy control for the three studied SNPs at both allele and genotype levels (*p*-value > 0.05). However, we reported that the ACC and ATC haplotypes had a significant association with schizophrenia (*p*-value 0.001). The ACC haplotype had more frequency in schizophrenia patients than the control group so that it could be considered a risk factor for schizophrenia, while the ATC haplotype was more frequent in control group and could be considered a protective haplotype.

The selected 3 SNPs are located in the promoter region of the FOXP3 gene. Rs3761548 is located at position −2383, rs3761549 is located at position −2383, and rs2232365 location is −924. The promoter region contains different DNA binding sites for transcription factors (TFs). Gene mutations in this region produce alterations in these binding sites and, by turn, affect TFs interaction with these sites, which affect FOXP3 gene expression either in quality or quantity [41]. AA genotype of rs3761548 leads to loss of binding sites for TFs E47 and C-Myb [34]. Moreover, the GG genotype for rs2232365 and TT genotype for rs3761549 lead to loss of binding site for GATA3, activating enhancer-binding protein 4 (AP4) TFs, respectively [26, 33]. The affection of FOXP3 protein in quality or quantity leads to Treg cell dysfunction and impairs the balance between Th1 and Th2 cells, disturbing the immune system’s balance toward the development of immunological and autoimmune diseases [41].

This study is one of few studies that focused on the role of FOXP3 in psychiatric disorders. There is a study from Iran on 523 autistic patients. The researchers tried to find an association between FOXP3 gene polymorphisms (rs3761548 and rs2232365) and an autism spectrum disorder. They observed a higher frequency of rs2232365 G allele and a significant association between rs2232365 GG genotype and autism in the dominant inheritance model. On the other hand, rs3761548 had no relation with the disease susceptibility. Also, on the level of haplotypes, no significant association was found [41].

The role of FOXP3 gene polymorphisms has been investigated in different disorders with an immunological background [20]. In Graves’ disease (GD), it was found that rs3761548 had no significant association with the disease in childhood and adolescence [7]; the same result was found in a study from the UK [35]. In the Japanese population, different results were obtained. A significant association with the development of the disease was detected [23]. Also, the A allele was found to be high risk for GD in Chinese patients [52]. For rs3761549, the GA genotype was significantly associated with GD in female patients in a Polish study [7], but no association was observed in another study [52].

ELSohafy et al. [15] found that the C allele and CC genotype of re3761548 are significantly increased in Egyptian patients with psoriasis. Another study investigated the role of rs3761548 and rs2232365. They found that AC genotype of rs3761548 was associated with psoriasis, but rs2232365 had no significant association with the disease [18]. Shen et al. [44] observed that the AA genotype of rs3761548 was associated with the disease. On the opposite to previous studies, Song et al. [45] found that both rs3761549 and rs2232365 were significantly associated with psoriasis, but rs3761548 had no association with the disease.

A study from Poland found no association between rs3761548 and rs3761549 gene polymorphisms and multiple sclerosis (MS) [48]. The same result was concluded in the Slovak population for rs3761548 [17] and also from the genome-wide association study (GWAS) on the Caucasian population [3]. Studies from Iran showed the opposite finding. Eftekharian et al. [14] detected a significant association between rs3761548 and 2232365 A allele and MS. The C-G and A-A haplotype blocks showed a significant association with MS [14]. Another study from Iran showed a significant association between AA and AC genotype for rs3761548 and the disease [25].

The FOXP3 SNPs (rs3761548, rs3761549, and rs2232365) were investigated in hematopoietic stem cell transplantation (HSCT) and renal transplantation. In HSCT, rs3761548 CC genotype was associated with veno-occlusive disease and cytomegalovirus (CMV) infection but no evidence of association with graft-versus-host disease (GVHD) or relapse [37]. Nam et al. [33], on the other hand, did not find an association with allogenic HSCT outcomes. In renal transplantation, decreased overall surveillance was associated with rs361548 and rs2232365 mutant genotypes compared with the wild one [32]. The rs3761548 AA genotype was associated with a fourfold increase in the risk of renal graft rejection, and in the same study, rs2232365 had no association [38]. During the 5-year follow-up after transplantation in patients receiving cyclosporin immunosuppressive drugs, the rs3761549 TT genotype was associated with rapid deterioration in renal functions [51].

We used the fixation index (Fst) to measure the proportion of genetic variation between subpopulations within the total population. The value of this index ranges from 0 to 1. The zero value means that two populations are breeding freely; in other words, there is a complete sharing of alleles. The one value means that the two populations do not share any alleles. We compared the alleles frequency of healthy controls in this study and the populations studied in the 1000 genome project (Table 6). This study’s healthy controls and the populations of the 1000 genome project showed similarity, but there was diversity between the Egyptian and the European populations for rs3761548.

They are accumulating pieces of evidence concerned with the FOXP3 gene and its role in the immune system, making it a candidate gene for studying the pathogenesis of schizophrenia. FOXP3 gene polymorphisms have been proved to be associated with different disorders with an immunological base due to alteration in TFs binding sites, which affect FOXP3 protein in both quantity and quality. Moreover, Treg cells have been studied in schizophrenia with contradictory results. A recent study investigated different T-cell populations in schizophrenia patients and showed that the number of natural Treg cells was increased in patients in unstimulated culture conditions, and the opposite happened in stimulated culture conditions [42]. Another study on patients with first-order psychosis showed an increased number of Treg cells [11]. However, opposite results were obtained in 18 treatment-resistant patients [16]. Deletion of FOXP3 leads to loss of Treg cell anti-inflammatory functions and its conversion to proinflammatory cells, but this action is still not proved in schizophrenia [49]. Although many immunological abnormalities have been reported in schizophrenia, the immune system’s role remains an unresolved issue. Whether immune system disturbance is the cause of schizophrenia or immunological abnormalities is secondary to the illness and sequel of genetics and environmental factors. Further evaluation of immune regulatory genes is recommended.

The main limitations of this study, which may contribute to false-positive or falsely negative results, are as follows: first, all populations of this study are from one locality. Second, the functions of FOXP3 and Treg cells in schizophrenia are still unclear and need more researches. Third, schizophrenia is a heterogeneous disorder with different clinical stages, which should be considered in future studies.

## Conclusion

No associations were found between any of the studied SNP genotypes and alleles. However, the ATC and ACC haplotypes showed statistically significant differences in the Egyptian population. We can consider this study as a light for the role of FOXP3 gene polymorphisms in schizophrenia. More extensive studies with multiple centers and regions’ involvement are recommended.

## Supplementary Information

**Additional file 1.**

## Data Availability

The datasets used and/or analyzed during the current study are available from the corresponding author on reasonable request.

## Abbreviations

Treg: Regulatory T cell
FOXP3: The forkhead box protein 3
PANSS: Positive and Negative Syndrome Scale
SNPs: Single-nucleotide polymorphisms
PCR-RFLP: Polymerase chain reaction-restriction fragments length polymorphism
MS: Multiple sclerosis
HSCT: Hematopoietic stem cell transplantation
TFs: Transcription factors
TH1: T helper 1
TH2: T helper 2
Fst: Fixation index
