# Supplementary material for: Analysis of selected polymorphisms in FOXP3 gene in a cohort of Egyptian patients with schizophrenia
Source: J Genet Eng Biotechnol. 2022 May 31;20:83. doi: 10.1186/s43141-022-00371-y (PMC9156649; doi:10.1186/s43141-022-00371-y)
Supplement: Supplementary file 1 — Additional file 1. [file 43141_2022_371_MOESM1_ESM.docx]

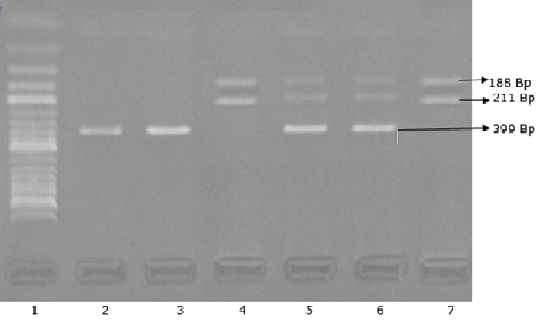


Figure 1: Typical band patterns of PCR-RFLP products for -3279C/A (rs3761548) Lane 2 and 3 indicated A/A genotype; Lane 4 and 7 indicated C/C genotype; Lane 5 and 6 indicated A/C genotype. Lane 1 indicates 50 Bp DNA marker


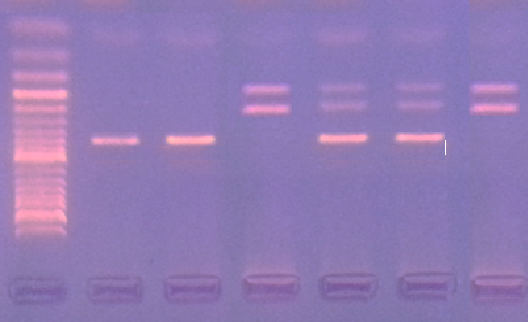


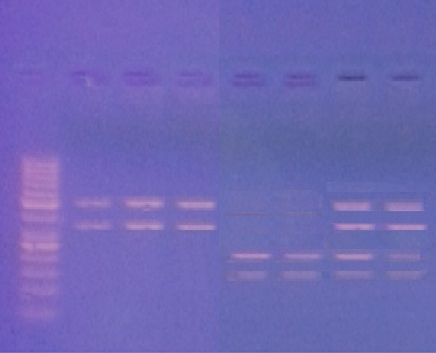


| 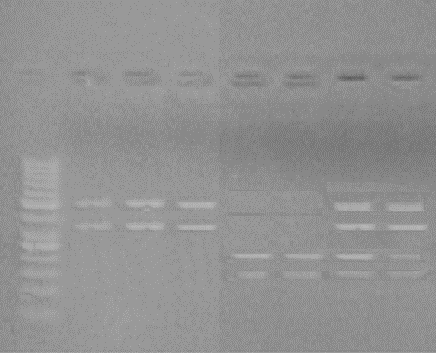 | 528 Bp  377 Bp  213 Bp  164 Bp |
| --- | --- |
| 1 2 3 4 5 6 7 8 |  |

Figure 2: Typical band patterns of PCR-RFLP products for -2383C/T (rs3761549) Lane 2, 3 and 4 indicated C/C genotype; Lane 5 and 6 indicated T/T genotype; Lane 7 and 8 indicated A/C genotype. Lane 1 indicates 50 Bp DNA marker


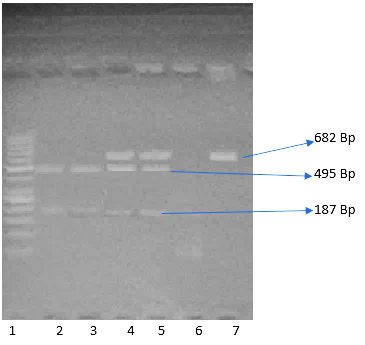


Figure 2: Typical band patterns of PCR-RFLP products for -924A/G (rs2232365) Lane 2 and 3 indicated G/G genotype; Lane 4 and 5 indicated A/G genotype; Lane 6 empty and 7 indicated A/A genotype. Lane 1 indicates 50 Bp DNA marker
